# Near-Field Source Localization in Nonuniform Noise: An Efficient Symmetric Matrix Factorization-Based Approach

**DOI:** 10.3390/s25185684

**Published:** 2025-09-12

**Authors:** Wenze Song, Zhenqing He, Guohao Sun, Shou Feng

**Affiliations:** 1 School of Aeronautics and Astronautics, Sichuan University, Chengdu 610065, China; sichuandxsongwz@163.com (W.S.); sgh2019@scu.edu.cn (G.S.); 2 Multi-Source Information Intelligent Fusion Key Laboratory of Sichuan Province, Chengdu 610065, China; 3 Robotic Satellite Key Laboratory of Sichuan Province, Chengdu 610015, China; 4 Southwest China Institute of Electronic Technology, Chengdu 610036, China; shoufeng_cetc@126.com

**Keywords:** near-field source localization, majorization–minimization, nonuniform noise, symmetric matrix factorization

## Abstract

This paper investigates the near-field source localization of multiple narrowband signals in the presence of unknown nonuniform noise with an arbitrary diagonal covariance matrix. From a covariance-fitting perspective, we reformulate the near-field localization problem as a joint symmetric matrix factorization and the estimation of nonuniform noise variances. This reformulation explicitly accounts for noise heterogeneity in the covariance structure, thereby avoiding noise mismodeling and enabling robust near-field localization for nonuniform noise. To solve the intractable symmetric matrix factorization problem, we develop a computationally efficient iterative algorithm based on the block majorization–minimization principle. The proposed algorithm has light per-iteration complexity and admits a closed-form iteration update. Furthermore, we also derive the Cramér–Rao bound (CRB) for near-field localization under nonuniform noise. Extensive numerical experiments demonstrate that the proposed approach outperforms the existing state-of-the-art near-field localization methods and closely matches the CRB while maintaining strong robustness against severe nonuniform noise.

## 1. Introduction

Source localization refers to extracting the spatial location parameters of one or more emitting sources through processing the observed signals acquired by a multi-antenna or multi-sensor array receiver. It enables a broad class of practical applications in array signal processing [1,2], wireless communications [3,4], radar systems [5,6], etc. Generally, according to the distance between the source and the receiving array, source localization can be classified into two typical cases: far-field and near-field. In the far-field case, where the source-to-array distance is much greater than $2D^{2}/\lambda$ (with *D* denoting the array aperture and λ the signal wavelength), the incident wavefront can be approximated as a planar wave, and the phase differences across sensors depend solely on the direction-of-arrival (DOA). Under such a plane-wave model, the localization task reduces to the well-studied DOA estimation problem, for which a variety of classical high-resolution algorithms are available, such as multiple signal classification (MUSIC) [7], maximum likelihood estimation (MLE) [8], estimation of signal parameters via rotational invariance techniques (ESPRIT) [9], etc.

Unlike the far-field case, the near-field scenario requires accounting for the spherical wavefront in modeling the electromagnetic propagation radiated by a point source [10,11]. The near-field spherical wavefront inherently carries both the range and angle information of the target, enabling the multi-sensor receiver to localize the target independently without assistance from multiple anchor devices. In recent years, the near-field localization problem has been extensively investigated. The most straightforward approach for near-field localization is to directly extend the far-field approach to the near-field scenario. Typical methods include the two-dimensional (2D) MUSIC algorithm [12] and the maximum likelihood-based approach [13], which involve an exhaustive 2D search over both range and angle and have high computational complexity. To avoid an exhaustive 2D search for the localization parameter and reduce the computational load, other popular methods focus on high-resolution subspace-based schemes that decouple the range and DOA estimation into separate one-dimensional searches [14,15,16,17].

However, the aforementioned near-field localization methods explicitly or implicitly assume that the sensor noises are spatially uniform (homogeneous) and that the corresponding diagonal noise covariance matrix shares a common variance. These methods fundamentally rely on the assumption of uniform noise and knowledge of the noise variance to accurately separate the signal subspace from the noise subspace. In the presence of nonuniform noise, however, the separation capability of the two subspaces can be severely degraded, since the unequal noise levels distort the orthogonality between the signal and noise subspaces, thereby leading to biased subspace estimation. In practical array systems, the assumption of homogeneous sensor noise is usually invalid, as noise powers can differ significantly across sensors due to various hardware and system nonidealities [18,19]. These influencing factors include nonuniform sensor responses, imperfections in the receiving channels, and mutual coupling effects, all of which contribute to spatial variations in noise characteristics across sensors. Therefore, the sensor noise in an array system should, in general, be modeled as the nonuniform noise with an arbitrary diagonal covariance matrix to fully characterize its spatial distribution.

When the spatially assumption of uniform noise is violated and the sensor noise is nonuniform, the (spectral) eigendecomposition of the sample covariance does not, in general, yield a noise subspace orthogonal to the array manifold. This loss of orthogonality undermines the projector-based criteria exploited by conventional near-field subspace methods, leading to biased parameter estimates and substantial degradation in localization accuracy. Currently, numerous approaches exist for far-field DOA estimation under nonuniform noise, including sparse signal representation-based methods [18] and iterative subspace-based schemes [20], which treat the sensor noise variances as unknown parameters to be estimated, thereby avoiding noise covariance mismodeling. To the best of our knowledge, the problem of near-field source localization in the presence of unknown nonuniform noise remains largely unexplored in the existing array processing literature. We note that in [21], a subspace-based localization method for near-field signals is developed under nonuniform noise with a symmetric uniform linear array (ULA). This approach employs the Toeplitz structure of the symmetric ULA correlation matrix to suppress the effect of nonuniform noise. However, its applicability is limited to ULAs and cannot be extended to other array configurations. In addition, it performs reliably only in regions with a high signal-to-noise ratio and fails to cope with highly nonuniform noise where the ratio between the maximum and minimum sensor noise powers is significantly large.

In this paper, we solve near-field source localization in the presence of nonuniform noise from the perspective of symmetric matrix factorization of the array output covariance matrix. Specifically, unlike the subspace-based scheme [21] which transforms the original nonuniform noise model into an equivalent uniform noise model, we reformulate the signal subspace estimation problem for near-field sources as a symmetric matrix factorization task from a covariance-fitting perspective, in which the unknown factor matrix and the nonuniform noise variances are jointly estimated. To solve the intractable non-convex symmetric matrix factorization problem, we develop an computationally efficient iterative algorithm based on inexact block coordinate descent, also referred to as block majorization–minimization [22,23]. The proposed algorithm has a low per-iteration computational complexity and admits a closed-form solution at each step. We further develop an accelerated version of the proposed algorithm by applying extrapolation. Then, we propose a reduced-dimension localization scheme to acquire the DOA and range estimations. Furthermore, we also derive the Cramér–Rao bound (CRB) for near-field localization in environments with nonuniform noise. Our extensive simulation results demonstrate that the proposed approach outperforms existing state-of-the-art near-field localization methods under nonuniform noise and closely approaches the CRB while also exhibiting strong robustness against severe nonuniform noise.

The remainder of this paper are organized as follows. Section 2 introduces the signal model. Section 3 presents the proposed symmetric matrix-factorization-based near-field localization approach. Section 4 derives the Cramér–Rao bound for near-field sources under nonuniform noise. Section 5 evaluates the performance of the proposed algorithm via extensive numerical simulations. Finally, Section 6 concludes the paper.

## 2. Signal Model

We consider *K* uncorrelated narrowband near-field sources impinging on a symmetric ULA (We consider the ULA structure to avoid a 2D search for localization as described in Section 3.3; however, the signal subspace acquisition via symmetric matrix factorization (introduced in Section 3.2) can be applicable to arbitrary array configurations) consisting of M=2N+1 isotropic sensors with uniform inter-element spacing *d*. The ULA structure is centered at the origin, with the central sensor indexed as 0 and the remaining sensors symmetrically labeled from −N to *N*, such that the leftmost and rightmost sensors correspond to indices −N and *N*, respectively. We use the parameter pair $\left(\theta_{k},r_{k}\right)$ to denote the DOA and the distance of the *k*-th (k=1,2,…,K) near-field signal. As such, the received baseband signal at the *m*-th sensor can be modeled as [17,24]

(1) $$z_{m}\left(t\right)=\sum_{k=1}^{K}s_{k}\left(t\right)e^{j(\gamma_{k}\left(m-N-1\right)+\phi_{k}{\left(m-N-1\right)}^{2})}+v_{m}\left(t\right),$$

where $\gamma_{k}=-2\pi d\sin\theta_{k}/\lambda$, $\phi_{k}=\pi d^{2}\cos^{2}\theta_{k}/\left(\lambda r_{k}\right)$, $j=\sqrt{-1}$ is the imaginary unit, λ is the signal wavelength, and $v_{m}\left(t\right)$ is the additive complex-valued Gaussian noise with zero mean and variance $\sigma_{m}^{2}$. As such, the M×1 output of the ULA receiver can be written as

(2) z(t)=A(θ,r)s(t)+v(t),

where $s\left(t\right)={\left[s_{1}\left(t\right),\ldots ,s_{K}\left(t\right)\right]}^{T}$ and $v\left(t\right)={\left[v_{1}\left(t\right),\ldots ,v_{M}\left(t\right)\right]}^{T}$ represent the source signal vector and the additive noise vector (in which ${\left(\cdot \right)}^{T}$ stands for the transpose operation), respectively. In addition, $A\left(\theta ,r\right)=[a\left(\theta_{1},r_{1}\right),\ldots ,a\left(\theta_{K},r_{K}\right)]$ is the near-field array manifold matrix, with its *k*-th steering vector being as follows:

(3) $$a\left(\theta_{k},r_{k}\right)={\left[e^{j(\gamma_{k}\left(-N\right)+\phi_{k}{\left(-N\right)}^{2})},\ldots ,1,\ldots ,e^{j(\gamma_{k}N+\phi_{k}N^{2})}\right]}^{T}.$$

Furthermore, we assume that the *K* signals ${\left\{s_{k}\left(t\right)\right\}}_{k=1}^{K}$ are temporally white with zero mean. The noise components ${\left\{n_{m}\left(t\right)\right\}}_{m=1}^{M}$ are statistically independent of all the signals ${\left\{s_{k}\left(t\right)\right\}}_{k=1}^{K}$. Then, we arrive at the following array output covariance matrix

(4) $$R=E\left\{z\left(t\right)z^{H}\left(t\right)\right\}=A\left(\theta ,r\right)PA^{H}\left(\theta ,r\right)+Q,$$

where E{·} and ${\left(\cdot \right)}^{H}$ denote the statistical expectation operator and the Hermitian transpose, respectively. Meanwhile, P and Q are the source signal covariance matrix and the noise covariance matrix, which are calculated as

(5) $$P=E\left\{s\left(t\right)s^{H}\left(t\right)\right\}=\mathrm{diag}\left\{p\right\},\;\;Q=E\left\{v\left(t\right)v^{H}\left(t\right)\right\}=\mathrm{diag}\left\{\sigma \right\},$$

respectively, where diag{·} indicates a diagonal matrix formed from a proper vector, $p={\left[p_{1}^{2},\ldots ,p_{K}^{2}\right]}^{T}$ is the source power vector, and $\sigma ={\left[\sigma_{1}^{2},\ldots ,\sigma_{M}^{2}\right]}^{T}$ is the noise power vector. The assumption of nonuniform noise means that Q is an arbitrary diagonal covariance matrix, i.e., the noise powers ${\left\{\sigma_{m}^{2}\right\}}_{m=1}^{M}$ are not necessarily identical across the array sensors, as a result of the nonidealities of the practical sensors.

The aim of this paper is to solve the problem of accurately estimating the *K* parameter pairs ${\left\{\left(\theta_{k},r_{k}\right)\right\}}_{k=1}^{K}$ of the near-field signals ${\left\{s_{k}\left(t\right)\right\}}_{k=1}^{K}$ in the presence of nonuniform noise and without any prior knowledge of the M=2N+1 noise powers ${\left\{\sigma_{m}^{2}\right\}}_{m=1}^{M}$.

## 3. Symmetric Matrix Factorization Based Near-Field Localization Approach

**Figure 1:**
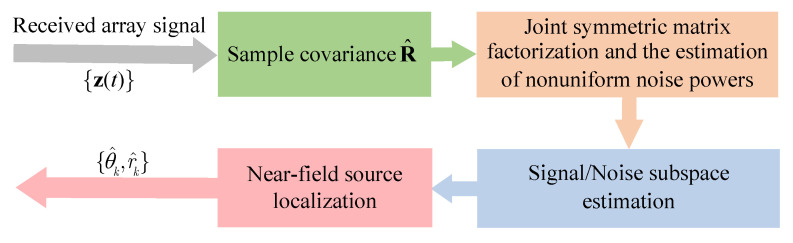
Flowchart of the proposed near-field localization approach.

This section presents the proposed approach for near-field localization under nonuniform noise. We first reformulate the near-field localization problem as a joint symmetric matrix factorization and the estimation of nonuniform noise powers. Second, we develop a computational iterative algorithm via inexact block coordinate descent to solve the symmetric matrix factorization problem. Then, we derive the near-field localization algorithm with a one-dimensional grid search. For clarity, a basic flowchart of the proposed near-field localization approach is illustrated in Figure 1, where $\hat{R}$ is the sample covariance matrix defined in (7), and $\left\{{\hat{\theta }}_{k},{\hat{r}}_{k}\right\}$ are the estimates of the true near-field location parameters $\left\{\theta_{k},r_{k}\right\}$. Finally, we derive the CRB for the near-field localization parameters under nonuniform noise.

### 3.1. Problem Reformulation

It is well known that the more accurate the subspace estimation, the better the localization performance. Traditional MUSIC-based schemes [7,12] directly apply the eigendecomposition to estimate the signal subspace or noise subspace. Under nonuniform noise, the separation capability between the signal subspace and the noise subspace can be severely degraded, since the unequal noise levels across antennas can destroy the orthogonality between the signal and noise subspaces. To this end, we consider a robust covariance-fitting criterion from a symmetric matrix factorization perspective.

Note that the full-rank signal covariance matrix P can be easily decomposed as follows: $P=LL^{H}$. As such, the output covariance matrix can be further expressed as

(6) $$\begin{array}{cc} R & =A\left(\theta ,r\right)PA^{H}\left(\theta ,r\right)+\mathrm{diag}\left\{\sigma \right\}\\ & =A\left(\theta ,r\right)LL^{H}A^{H}\left(\theta ,r\right)+\mathrm{diag}\left\{\sigma \right\}\\ \; & =XX^{H}+\mathrm{diag}\left\{\sigma \right\}, \end{array}$$

where $X=AL\in C^{M\times K}$ has the same column space as the true array manifold matrix A(θ,r). This implies that we can acquire the signal or noise subspace by solving a symmetric matrix factorization problem to estimate the full-rank factor matrix X.

In practice, only a finite number of *T* snapshots are available, and the sample covariance matrix is used as an estimate of the ideal R:

(7) $$\hat{R}=\frac{1}{T}\sum_{t=1}^{T}z\left(t\right)z^{H}\left(t\right).$$

**Figure 2:**
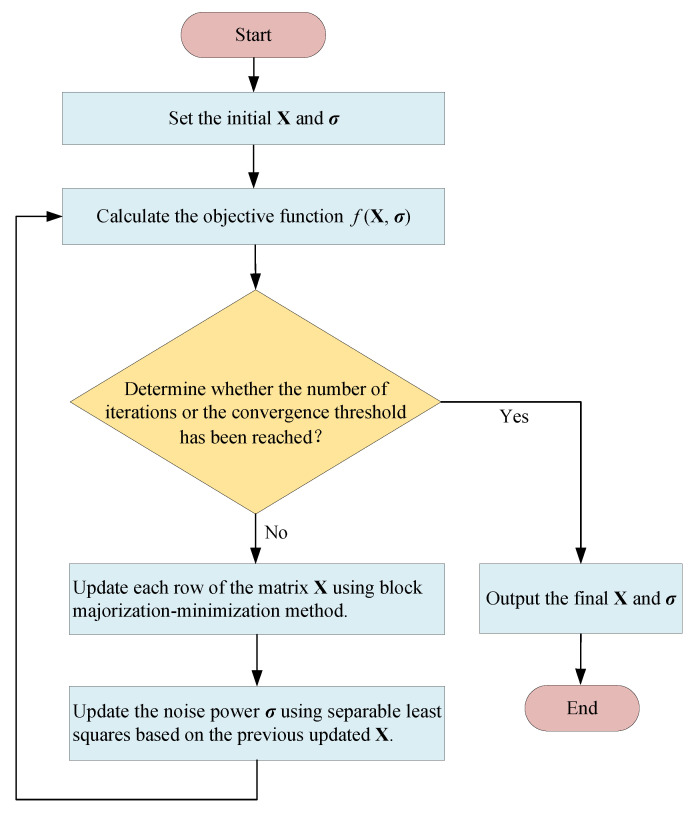
Flow diagram for solving the symmetric matrix factorization problem.

With the available observation covariance matrix $\hat{R}$(≈R), we arrive at the following symmetric matrix factorization problem:

(8) $$\min_{X,\sigma }f\left(X,\sigma \right)≜{∥\hat{R}-XX^{H}-\mathrm{diag}\left\{\sigma \right\}∥}_{F}^{2},$$

where ${∥\cdot ∥}_{F}$ denotes the Frobenius norm. Notice that problem (8) is essentially a covariance matrix-fitting problem with the symmetric matrix factorization $XX^{H}$ and an unknown noise variance vector σ. We see that such a reformulation explicitly accounts for noise heterogeneity in the covariance structure, thereby avoiding noise mismodeling and enabling robust subspace estimation. In addition, the symmetric matrix factorization formulation (8) enables the design of a computationally efficient iterative algorithm, as will be elaborated in the following subsection, based on block majorization–minimization, where each iteration admits an elegant closed-form update. For ease of understanding, we illustrate the flow diagram for solving the symmetric matrix factorization problem (8) in Figure 2.

### 3.2. Inexact Block Coordinate Descent Algorithm for Symmetric Matrix Factorization

To solve problem (8), we employ the inexact block coordinate descent scheme, usually called block majorization–minimization [22,23]. The core idea is to decompose the original multi-variable non-convex problem into a series of relatively simple subproblems with a closed-form solution through alternating optimization. The main motivation for employing block majorization–minimization to solve problem (8) is that each alternating subproblem entails low iteration complexity, admits an elegant closed-form update, and does not require a complicated line-search procedure as in gradient descent schemes. Specifically, the factor matrix X is updated in a row-by-row manner, and subsequently, the noise variance vector σ is refined according to the previous updated X.

First, we discuss how to update each row of X. Let us take the example of updating the *i*-th row, denoted by x, of X, while all other variables, including the remaining rows of X and σ, are fixed at the previous iteration. As such, the intermediate matrix product of the factor matrix X in Equation (8) can be expressed as

(9) $$XX^{H}=\left(\begin{array}{c} {\bar{X}}_{i}\\ x^{H}\\ {\underline{X}}_{i} \end{array}\right){\left(\begin{array}{c} {\bar{X}}_{i}\\ x^{H}\\ {\underline{X}}_{i} \end{array}\right)}^{H}=\left(\begin{array}{ccc} {\bar{X}}_{i}{\bar{X}}_{i}^{H} & {\bar{X}}_{i}x^{H} & {\bar{X}}_{i}{\underline{X}}_{i}^{H}\\ x^{H}{\bar{X}}_{i}^{H} & x^{H}x & x^{H}{\underline{X}}_{i}^{H}\\ {\underline{X}}_{i}{\bar{X}}_{i}^{H} & {\underline{X}}_{i}x^{H} & {\underline{X}}_{i}{\underline{X}}_{i}^{H} \end{array}\right).$$

Moreover, to simplify the objective function, we define the partitioned components as

(10) $$\begin{array}{cc} {\bar{X}}_{i} & ≜X_{1:i-1,:}, \end{array}$$

(11) $$\begin{array}{cc} {\underline{X}}_{i} & ≜X_{i+1:2N+1,:}, \end{array}$$

(12) $$\begin{array}{cc} M & =R-\mathrm{diag}\{\sigma \}, \end{array}$$

(13) $$\begin{array}{cc} {\bar{m}}_{i} & ≜M_{1:i-1,i},\;\;{\underline{m}}_{i}≜M_{i+1:2N+1,i}, \end{array}$$

where $X_{1:i-1,:}$ is the submatrix of matrix X, consisting of the first row to the i−1-row; $X_{i+1:2N+1,:}$ is the submatrix of matrix X, consisting of the i+1-row to the 2N+1-row; $M_{1:i-1,i}$ is the submatrix of matrix M, consisting of the 1-th row to the i−1 row and the *i*-th column; and $M_{i+1:2N+1,i}$ is the submatrix of matrix M, consisting of the i+1 row to the 2N+1-th row and the *i*-th column.

By ignoring terms irrelevant to the *i*-th row and letting $\rho_{ij}$ denote the (i,j)-th element of matrix M. The corresponding minimization problem for the *i*-th (1≤i≤2N+1) row can be formulated as

(14) $$X_{i:}^{H}=\arg\min_{x}\;2\left(∥{\bar{m}}_{i}-{\bar{X}}_{i}{x∥}^{2}+{∥{\underline{m}}_{i}-{\underline{X}}_{i}x∥}^{2}\right)+{\left|\rho_{ii}-{∥x∥}^{2}\right|}^{2}.$$

The above subproblem can be further denoted as

(15) Xi:H=argminx∥x∥4+2xHVix−4ReX¯iHm¯i+X_iHm_ix,

where $V_{i}≜\Psi_{i}-\rho_{ii}I$ and $\Psi_{i}≜{\underline{X}}_{i}^{H}{\underline{X}}_{i}+{\bar{X}}_{i}^{H}{\bar{X}}_{i}$. It is known that, due to the Lipschitz continuity of the quadratic term ${\bar{x}}^{H}V_{i}x$, an upper bound for the subobjective function of problem (15) can be obtained. Specifically, by introducing a Lipschitz continuity constraint and adding a regularization term, the quadratic term of the subobjective function can be approximated as follows:

(16) $$x^{H}V_{i}x\leq y^{H}V_{i}y+2y^{H}V_{i}\left(x-y\right)+S{∥x-y∥}^{2},\;\forall x,y,$$

where *S* denotes the Lipschitz constant for the quadratic form $x^{H}V_{i}x$, which can be set as $\max\left(\Psi_{i}I\right)-\rho_{ii}$, i.e., the associated maximum eigenvalue.

By replacing $x^{H}V_{i}x$ in Equation (15) with the above upper bound evaluated at $\tilde{x}≜{\tilde{X}}_{i:}^{H}$ (i.e., let $y=\tilde{x}$ and the current X is denoted as $\tilde{X}$) and rearranging the terms, we arrive at

(17) Xi:H=argminx∥x∥4+2S∥x∥2−4RebiHx,

where $b_{i}=-V_{i}\tilde{x}+S\tilde{x}+\kappa_{i}$ with $\kappa_{i}={\bar{X}}_{i}^{H}{\bar{m}}_{i}+{\underline{X}}_{i}^{H}{\underline{m}}_{i}$. The analytical solution to the above problem (17) is given by [25]

(18) $$X_{i:}^{H}=\eta \frac{b_{i}}{∥b_{i}∥},$$

where $\eta =\sqrt[{3}]{\frac{∥b_{i}∥}{2}-\sqrt{\Delta }}+\sqrt[{3}]{\frac{∥b_{i}∥}{2}+\sqrt{\Delta }}$ with $\Delta ≜\frac{∥b_{i}{∥}^{2}}{4}+\frac{S^{3}}{27}$.

We now update the noise power vector σ. With X being fixed, the unknown variable in the objective function f(X,σ) only includes σ. It is not difficult to see that the corresponding minimization problem is separable for each element of σ and then the updated noise powers can be calculated as follows

(19) $$\sigma =\mathrm{diag}(\hat{R}-XX^{H}).$$

The above closed-form updates in (18) and (19) can be accelerated by employing extrapolation [26]. For instance, we elaborate the accelerated update to x as follows. Let the *i*-th row of the original matrix X before the previous iteration be denoted as ${\left(X_{i:}^{H}\right)}^{w}$, and the updated vector for the *i*-th row obtained in the current iteration be ${\left(X_{i:}^{H}\right)}^{w+1}$. We can further obtain the accelerated update as follows [26]:

(20) $$\tau^{w+1}={\left(X_{i:}^{H}\right)}^{w+1}+\alpha_{w+1}\left[{\left(X_{i:}^{H}\right)}^{w+1}-{\left(X_{i:}^{H}\right)}^{w}\right],$$

where $\tau^{w+1}$ replaces the *i*-th row of ${\left(X_{i:}^{H}\right)}^{w}$ of X, and $\alpha_{w+1}=\frac{l_{k}-1}{l_{k+1}}$, $l_{k+1}=\frac{1+\sqrt{1+4l_{k}^{2}}}{2}$, and $l_{0}=1$. For the accelerated update to σ, we can adopt the same rule as that for the the accelerated update to x. Finally, by repeating the entire procedure on x and σ until a convergence criterion is met, we can obtain a final estimate of the factor matrix X.

### 3.3. Near-Field Localization Algorithm

To extract the signal subspace and noise subspace from the obtained factor matrix X, we employs the orthogonal–triangular (QR) decomposition. First, a reduced QR decomposition is performed on matrix X, yielding $X=Q^{\prime }R^{\prime }$, where $Q^{\prime }$ has orthogonal columns and $R^{\prime }$ is an upper triangular matrix. Subsequently, the signal subspace basis $U_{s}$ is constructed by selecting the first *K* columns of $Q^{\prime }$. To obtain the noise subspace $U_{n}$, the signal subspace $U_{s}$ is extended to a complete orthonormal basis by constructing the full orthogonal matrix $Q^{\prime }=\left[U_{s}|U_{n}\right]$ through QR decomposition. This decomposition achieves effective separation of the signal and noise subspaces.

Although exact localization information can be obtained through a 2D search using the estimated noise or signal subspaces, this approach suffers from localization inaccuracy and high computational complexity. To address these limitations, we propose a reduced-dimension localization scheme with a one-dimensional search, which benefits from the symmetric ULA structure, to acquire the DOA and range estimations. It is worth noting that the symmetric matrix factorization algorithm introduced in the above subsection for signal subspace estimation can be used for arbitrary array configurations such as circular array and rectangular array. This is because the subspace estimation does not depend on the special array configuration.

The near-field steering vector a(θ,r) in Equation (3) can be decomposed into the product of two matrices, Γ(γ) and B(ϕ), corresponding to angular and range parameters, respectively. This decomposition can be expressed as

(21) a(θ,r)=Γ(γ)B(ϕ),

where

$$\Gamma \left(\gamma \right)=\left[\begin{array}{cccc} e^{j(-N)\gamma } & & \\ & e^{j(-N+1)\gamma } & \\ & & \ddots \\ & & & 1\\ & & \begin{array}{c} ⋰ \end{array}\\ & e^{j(N-1)\gamma } & \\ e^{jN\gamma } & \end{array}\right],$$

$$B\left(\phi \right)=\left[\begin{array}{c} e^{j{\left(-N\right)}^{2}\phi }\\ e^{j{\left(-N+1\right)}^{2}\phi }\\ \vdots \\ e^{j{\left(-1\right)}^{2}\phi }\\ 1 \end{array}\right],$$

with $a\left(\theta ,r\right)\in C^{\left(2N+1\right)}$, and *N* being the number of signal-receiving elements.

Note that the spatial spectrum function is reconstructed as the inverse of the 2D-MUSIC pseudospectrum shown below:

(22) $$E\left(\theta ,r\right)=B^{H}\left(\phi \right)O\left(\gamma \right)B\left(\phi \right),$$

where $O\left(\gamma \right)=\Gamma^{H}\left(\gamma \right)U_{n}U_{n}^{H}\Gamma \left(\gamma \right)$ denotes the transformed noise subspace matrix. This formulation leads to the following constrained optimization problem:

(23) $$\min_{\gamma ,\;\phi }B^{H}\left(\phi \right)O\left(\gamma \right)B\left(\phi \right)\;\mathrm{subject}\;\mathrm{to}\;e^{H}B\left(\phi \right)=1,$$

with $e={\left[0,0,\cdots ,0,1\right]}^{T}\in R^{\left(N+1\right)}$ being a selection vector. To solve the above constrained problem, we construct the Lagrangian function as follows:

(24) $$L\left(\gamma ,\phi \right)=B^{H}\left(\phi \right)O\left(\gamma \right)B\left(\phi \right)-\varepsilon \left(e^{H}B\left(\phi \right)-1\right),$$

where ε represents the Lagrange multiplier. By taking the derivative of the above Lagrangian function and performing identity transformations, we can eliminate the Lagrange multiplier ε and obtain

(25) $$B\left(\phi \right)=\frac{O^{-1}\left(\gamma \right)e}{e^{H}O^{-1}\left(\gamma \right)e}.$$

Combining (23) and (25), the optimization problem reduces to

(26) $${\hat{\gamma }}_{k}=\arg\max_{\gamma }e^{H}O^{-1}\left(\gamma \right)e.$$

This formulation requires only a single 1D spectral search, instead of the 2D search. The corresponding vector is computed as follows:

(27) $$\hat{B}\left(\phi_{k}\right)=\frac{O^{-1}\left({\hat{\gamma }}_{k}\right)e}{e^{H}O^{-1}\left({\hat{\gamma }}_{k}\right)e}.$$

Then, the phase components are extracted via

(28) $${\hat{g}}_{k}=\mathrm{angle}\left(\hat{B}\left(\phi_{k}\right)\right)=G\phi_{k},\;G={\left[0,1,\cdots ,N^{2}\right]}^{T},$$

where angle(·) represents the phase. The parameter $\phi_{k}$ is estimated by using least squares. The least-squares solution is given by

(29) $${\hat{c}}_{k}={\left(\varpi^{T}\varpi \right)}^{-1}\varpi^{T}{\hat{g}}_{k},\;c_{k}=\left[\begin{array}{c} c_{k0}\\ {\hat{\phi }}_{k} \end{array}\right],\;\varpi =\left[\begin{array}{cc} 1 & 0\\ 1 & 1\\ \vdots & \vdots \\ 1 & N^{2} \end{array}\right],$$

where $c_{k0}$ compensates for the phase offset errors. Eventually, the estimates of DOA and range are given by

(30) $${\hat{\theta }}_{k}=-\arcsin\left(\frac{{\hat{\gamma }}_{k}\lambda }{2\pi d}\right),\;{\hat{r}}_{k}=\frac{\pi d^{2}}{\lambda {\hat{\phi }}_{k}}\cos^{2}{\hat{\theta }}_{k},$$

where λ denotes wavelength and *d* element spacing.

Based on the algorithmic descriptions for subspace estimation and near-field parameter estimation in the above two subsections, we provide the pseudocode for the proposed near-field localization algorithm in Algorithm 1. The proposed algorithm consists of two stages. The first stage iteratively updates X to capture the symmetric matrix factor. The second stage uses optimized X for near-field localization: QR decomposition is applied to extract signal or noise subspaces, followed by a 1D MUSIC spectral search and least-squares fitting to estimate the DOAs and ranges of all source signals.

### 3.4. Computational Complexity Analysis

Now, we conduct a computational complexity analysis of the proposed near-field localization algorithm. First, we analyze the computational complexity of the symmetric matrix factorization to obtain the updated factor matrix X, which corresponds to Lines 1–21 of Algorithm 1. The main computational complexity of the symmetric matrix factorization algorithm involves the matrix–vector calculations in Lines 7–10 of Algorithm 1. In line 7, since ${\bar{X}}_{i}^{H}\in C^{K\times (i-1)}$ and $X_{i}^{H}\in C^{K\times M}$, the computational complexity is $O(\left(i-1\right)K^{2}+\left(M-i\right)K^{2})$, and since i<M, it can be reduced to $O(MK^{2})$. Similarly, the computational complexity of calculating the matrix $V_{i}$ is O(K). The complexity of performing eigenvalue decomposition on a K×K matrix $\Psi_{i}$ is $O(K^{3})$. Since $\bar{m_{i}}\in C^{(i-1)\times i},\underline{m_{i}}\in C^{(M-i)\times i}$, we can obtain the computational complexity required to calculate the matrix $b_{i}$ as follows: $O\left(K^{3}\right)+O\left(MK^{2}\right)+O\left(K\right)+O\left(MK\right)=O\left(K^{3}\right)+O\left(MK^{2}\right)$. In Lines 11–17 each line has a computational complexity of O(K). Therefore, for a single loop in Lines 5–18, the overall computational complexity is $O\left(K^{3}\right)+O\left(MK^{2}\right)+O\left(K\right)=O\left(K^{3}\right)+O\left(MK^{2}\right).$

After *M* successive loops, the computational complexity of Lines 5–18 is $O\left(MK^{3}\right)+O\left(M^{2}K^{2}\right)$. Line 4 of Algorithm 1 performs *M* addition operations, while the complexity of Line 19 is $M^{2}\left(K+T\right)$ flops. Therefore, we ultimately obtain the computational complexity of Lines 1–21 of Algorithm 1 as follows:

(31) $$O\left(W_{\max}MK^{3}\right)+O\left(W_{\max}M^{2}\left(K^{2}+T\right)\right)+O\left(W_{\max}M\right),$$

where $W_{\max}$ denotes the maximum number of iterations for the symmetric matrix factorization algorithm.
**Algorithm 1** Proposed Algorithm Procedure for Near-field Localization**Input:** $R\in C^{M\times M}$, *K*1:Initialize $X^{\left(0\right)}\in C^{M\times M}$, iteration counter w←02:Initialize $\mathrm{diag}{\left\{\sigma \right\}}^{\left(0\right)}=\mathrm{diag}\left(\hat{R}-X^{\left(0\right)}\right)$3:**repeat**4:   $M=\hat{R}-\mathrm{diag}{\left\{\sigma \right\}}^{\left(w\right)}$, where ${\left(\cdot \right)}^{\left(w\right)}$ represents the matrix in the *w*th iteration.5:   **for** i=1 to *M* **do**6:     ${\bar{m}}_{i}=M_{1:i-1,i}$ and ${\underline{m}}_{i}=M_{i+1:M,i}$7:     $\Psi_{i}≜{\underline{X}}_{i}^{H}{\underline{X}}_{i}+{\bar{X}}_{i}^{H}{\bar{X}}_{i}$8:     Set $V_{i}=\Psi_{i}-\rho_{ii}I$ and $S=\max\left(\mathrm{eig}\left(\Psi_{i}\right)\right)-\rho_{ii}$9:     $\kappa_{i}={\bar{X}}_{i}^{H}{\bar{m}}_{i}+{\underline{X}}_{i}^{H}{\underline{m}}_{i}$10:     $b_{i}=-V_{i}x^{\left(w\right)}+Sx^{\left(w\right)}+\kappa_{i}$ where $x^{\left(w\right)}={\left(X_{i:}^{H}\right)}^{\left(w\right)}$11:     $\Delta =\frac{∥b_{i}{∥}^{2}}{4}+\frac{S^{3}}{27}$12:     $\eta =\sqrt[{3}]{\frac{∥b_{i}∥}{2}+\sqrt{\Delta }}+\sqrt[{3}]{\frac{∥b_{i}∥}{2}-\sqrt{\Delta }}$13:     ${\left(X_{i:}^{H}\right)}^{\left(w+1\right)}=\eta \frac{b_{i}}{∥b_{i}∥}$14:     $l_{k+1}=\frac{1+\sqrt{1+4l_{k}^{2}}}{2}$ with $l_{0}=1$15:     $\alpha_{w+1}=\frac{l_{k-1}}{l_{k+1}}$16:     $\tau^{w+1}={\left(X_{i:}^{H}\right)}^{w+1}+\alpha_{w+1}\left[{\left(X_{i:}^{H}\right)}^{w+1}-{\left(X_{i:}^{H}\right)}^{w}\right]$17:     ${\left(X_{i:}^{H}\right)}^{\left(w+1\right)}=\tau^{w+1}$18:   **end for**19:   $\mathrm{diag}{\left\{\sigma \right\}}^{\left(w+1\right)}=\mathrm{diag}\left(\hat{R}-X^{\left(w+1\right)}{\left(X^{\left(w+1\right)}\right)}^{H}\right)$20:   w←w+121:**until** a convergence criterion is met.22:$\left[Q^{\prime },\;R^{\prime }\right]=\mathrm{qr}\left(X\right)$23:$U_{s}=Q^{\prime }\left(:,1:K\right),\;\;U_{n}=Q^{\prime }\left(:,K+1:M\right)$24:**for**k=1 to *K* **do**25:   a(γ,ϕ)=Γ(γ)B(ϕ)26:   Perform 1D MUSIC spectral search over ϕ using (22)27:   Find the optimal ${\hat{\gamma }}_{k}$ in (26) and compute $\hat{B}\left(\phi_{k}\right)=\frac{O^{-1}\left({\hat{\gamma }}_{k}\right)e}{e^{H}O^{-1}\left({\hat{\gamma }}_{k}\right)e}$28:   ${\hat{g}}_{k}=\mathrm{angle}\left(\hat{B}\left(\phi_{k}\right)\right)=G\phi_{k}$, where $G={\left[0,1,\ldots ,N^{2}\right]}^{T}$29:   $\varpi =\left[\begin{array}{cc} 1 & 0\\ 1 & 1\\ \vdots & \vdots \\ 1 & N^{2} \end{array}\right]$30:   ${\hat{c}}_{k}=\left[\begin{array}{c} c_{k0}\\ {\hat{\phi }}_{k} \end{array}\right]={\left(\varpi^{T}\varpi \right)}^{-1}\varpi^{T}{\hat{g}}_{k}$31:   ${\hat{\theta }}_{k}=-\arcsin\left(\frac{{\hat{\gamma }}_{k}\lambda }{2\pi d}\right)$32:   ${\hat{r}}_{k}=\frac{\pi d^{2}}{\lambda {\hat{\phi }}_{k}}\cos^{2}{\hat{\theta }}_{k}$33:**end for****Output:**${\left\{\left({\hat{\theta }}_{k},{\hat{r}}_{k}\right)\right\}}_{k=1}^{K}$

For the near-field localization algorithm, we analyze its computational complexity as follows: The computational complexity of the proposed algorithm is dominated by the one-dimensional spectral search procedure, which is executed for $n_{g}$ grid points. For each candidate value $\gamma_{i}$, the algorithm constructs the matrix $O\left(\gamma_{i}\right)=\Gamma^{H}\left(\gamma_{i}\right)U_{n}U_{n}^{H}\Gamma \left(\gamma_{i}\right)$, which involves two sequential matrix multiplications requiring $O(M^{2}\left(M+1\right))$ and $O(M{\left(M+1\right)}^{2})$ flops, respectively. The subsequent matrix inversion $O^{-1}\left(\gamma_{i}\right)$ needs $O({\left(M+1\right)}^{3})$ flops, followed by the quadratic form evaluation $e^{H}O^{-1}\left(\gamma_{i}\right)e$ with $O({\left(M+1\right)}^{2})$ complexity. Thus, the total computational complexity can be expressed as

(32) $$O\left(n_{g}\left(M+1\right)\left[\frac{\left(M-K)(3M+1\right)}{4}+\frac{{\left(M+1\right)}^{2}}{8}\right]\right),$$

where the first term (M−K)(3M+1)/4 represents the complexity of noise subspace computation, with the factor 3/4 arising from exploiting the Hermitian structure of $U_{n}U_{n}^{H}$, and the second term ${\left(M+1\right)}^{2}/8$ accounts for matrix inversion and quadratic form evaluations, with the factor 1/8 resulting from combining these operations and utilizing the positive definite property of O(γ). Therefore, the computational complexity of the proposed algorithm is ultimately expressed as

(33) $$O\left(n_{g}\left(M+1\right)\left[\frac{\left(M-K)(3M+1\right)}{4}+\frac{{\left(M+1\right)}^{2}}{8}\right]+W_{\max}\left[MK^{3}+M^{2}\left(K^{2}+T\right)+M\right]\right).$$

Additionally, we list the computational complexities of the proposed algorithm, MUSIC [7], Capon [27], and RD-MUSIC [17] for comparison in Table 1, where $n_{g_{\theta }}$ is the time of search for theta in the 2D-MUSIC algorithm, $n_{g_{r}}$ is the time of search for range in the 2D-MUSIC algorithm, and $N_{s}$ is the number of spatial search points. As shown in Table 1, the proposed algorithm achieves significantly lower computational complexity. Specifically, for large *M*, its dominant term scales as $O(M^{2})$, whereas the competing methods, RD-MUSIC, Capon, and MUSIC, all involve cubic-order operations in the order of $O(M^{3})$. This indicates that the proposed method exhibits superior scalability for large-scale array scenarios. In addition, the practical real-time trade-offs between accuracy and computational cost can be achieved by tuning the grid resolution, the number of snapshots, or the maximum number of inner iterations, which makes the proposed method both computationally efficient and flexible for practical deployment.

## 4. CRB for Near-Field Sources in Nonuniform Noise

As a fundamental benchmark for evaluating the performance of an estimator, the Cramér–Rao bound (CRB) provides a theoretical lower bound on the error variance of any unbiased estimator. We derive the stochastic CRB for the near-field localization problem in the presence of nonuniform noise. Although the near-field CRB was derived in [21], it remains complex and cannot be applied to cases where the number of sources exceeds the number of sensors. In this paper, we derive a closed-form CRB in a concise manner by exploiting the property of the Khatri–Rao product. In the considered near-field localization scenario with nonuniform noise, we collectively denote all unknown parameters as

(34) $$\psi ={\left[\theta^{T},r^{T},\delta^{T}\right]}^{T},\;\delta^{T}={\left[p^{T},\sigma^{T}\right]}^{T}.$$

Recall that the CRB is defined as the inverse of the Fisher information matrix (FIM). Consequently, according to the relation $\mathrm{trace}\left\{\mathrm{WXYZ}\right\}={\mathrm{vec}}^{H}\left(X^{H}\right)\left(W^{T}⊗Y\right)\mathrm{vec}\left(Z\right)$, where ⊗ stands for the Kronecker product, the (q,l)-th entry of the FIM associated with the output covariance matrix R is computed as follows [18,28,29]:

(35) $$\begin{array}{cc} {\left[F\right]}_{q,l} & =T\;\mathrm{Trace}\left\{R^{-1}\frac{\partial R}{\partial \psi_{q}}R^{-1}\frac{\partial R}{\partial \psi_{l}}\right\}\\ \; & =T\;r_{q}^{H}W_{R}r_{l},\;\;q,l=1,\ldots ,3K+M, \end{array}$$

where $r_{q}=\mathrm{vec}\left(\partial R/\partial \psi_{q}\right)$, and $W_{R}=R^{-T}⊗R^{-1}$, $\psi_{q}$ is the *q*-th entry of the parameter ψ. As such, the total FIM can be compactly written as

(36) $$F=\left[\begin{array}{ccc} F_{\theta \theta } & F_{\theta r} & F_{\theta \delta }\\ F_{r\theta } & F_{rr} & F_{r\delta }\\ F_{\delta \theta } & F_{\delta r} & F_{\delta \delta } \end{array}\right].$$

With the signal model assumption introduced in Section 2, the output covariance matrix R in Equation (4) can be further expressed as

(37) $$R=\sum_{k=1}^{K}a\left(\theta_{k},r_{K}\right)p_{k}^{2}a^{H}\left(\theta_{k},r_{k}\right)+\sum_{m=1}^{M}\sigma_{m}^{2}\vartheta_{m}\vartheta_{m}^{T},$$

where $\vartheta_{m}\in C^{M}$ is the canonical basis vector, with its *m*-th element being 1. Combining (37) and $\mathrm{vec}({\mathrm{ba}}^{T})=a⊗b$, the vector $r_{q}$ used for the FIM of all parameters in Equation (35) can be calculated as follows:

(38) $$\begin{array}{cc} r_{\theta_{k}} & =\mathrm{vec}\left(\frac{\partial R}{\partial \theta_{k}}\right)=p_{k}^{2}\mathrm{vec}\left[\frac{\partial a(\theta_{k},r_{k})}{\partial \theta_{k}}a^{H}\left(\theta_{k},r_{k}\right)+a\left(\theta_{k},r_{k}\right){\left(\frac{\partial a(\theta_{k},r_{k})}{\partial \theta_{k}}\right)}^{H}\right] \end{array}$$

(39) $$\begin{array}{cc} \; & =p_{k}^{2}\left[a^{*}\left(\theta_{k},r_{k}\right)⊗\frac{\partial a(\theta_{k},r_{k})}{\partial \theta_{k}}+{\left(\frac{\partial a(\theta_{k},r_{k})}{\partial \theta_{k}}\right)}^{*}⊗a\left(\theta_{k},r_{k}\right)\right], \end{array}$$

(40) $$\begin{array}{cc} r_{r_{k}} & =\mathrm{vec}\left(\frac{\partial R}{\partial r_{k}}\right)=p_{k}^{2}\mathrm{vec}\left[\frac{\partial a(\theta_{k},r_{k})}{\partial r_{k}}a^{H}\left(\theta_{k},r_{k}\right)+a\left(\theta_{k},r_{k}\right){\left(\frac{\partial a(\theta_{k},r_{k})}{\partial r_{k}}\right)}^{H}\right] \end{array}$$

(41) $$\begin{array}{cc} \; & =p_{k}^{2}\left[a^{*}\left(\theta_{k},r_{k}\right)⊗\frac{\partial a(\theta_{k},r_{k})}{\partial r_{k}}+{\left(\frac{\partial a(\theta_{k},r_{k})}{\partial r_{k}}\right)}^{*}⊗a\left(\theta_{k},r_{k}\right)\right], \end{array}$$

(42) $$\begin{array}{cc} r_{p_{k}^{2}} & =\mathrm{vec}\left(\partial R/\partial p_{k}^{2}\right)=\mathrm{vec}\left[a\left(\theta_{k},r_{k}\right)a^{H}\left(\theta_{k},r_{k}\right)\right]=a^{*}\left(\theta_{k},r_{k}\right)⊗a\left(\theta_{k},r_{k}\right), \end{array}$$

(43) $$\begin{array}{cc} r_{\sigma_{m}^{2}} & =\mathrm{vec}\left(\partial R/\partial \sigma_{m}^{2}\right)=\mathrm{vec}\left(\vartheta_{m}\vartheta_{m}^{T}\right)=\vartheta_{m}⊗\vartheta_{m}, \end{array}$$

where ${\left(\cdot \right)}^{*}$ stands for the complex conjugate operation. Now, armed with (35) and (43), we calculate the submatrices of F in Equation (36) as follows:

(44) $$\begin{array}{cc} \; & F_{\theta \theta }=T\;D_{\theta }^{H}W_{R}D_{\theta },F_{rr}=T\;D_{r}^{H}W_{R}D_{r}, \end{array}$$

(45) $$\begin{array}{cc} \; & F_{\delta \delta }=T\;D_{\delta }^{H}W_{R}D_{\delta },F_{\theta \delta }=T\;D_{\theta }^{H}W_{R}D_{\delta }, \end{array}$$

(46) $$\begin{array}{cc} \; & F_{\theta r}=T\;D_{\theta }^{H}W_{R}D_{r},F_{r\delta }=T\;D_{r}^{H}W_{R}D_{\delta }, \end{array}$$

where $F_{\delta \theta }=F_{\theta \delta }^{H},F_{r\theta }=F_{\theta r}^{H},F_{\delta r}=F_{r\delta }^{H}$. The corresponding terms employed in the computation of the above sub-FIMs are given by

(47) $$\begin{array}{cc} \; & D_{\theta }=\left[r_{\theta_{1}},\ldots ,r_{\theta_{K}}\right]=\left[A_{1}^{*}\left(\theta ,r\right)⊙A_{1}^{\prime }\left(\theta ,r\right)+{\left(A_{1}^{\prime }\left(\theta ,r\right)\right)}^{*}⊙A_{1}\left(\theta ,r\right)\right]P, \end{array}$$

(48) $$\begin{array}{cc} \; & A_{1}^{\prime }\left(\theta ,r\right)=\left[\frac{\partial a(\theta_{1},r_{1})}{\partial \theta_{1}},\ldots ,\frac{\partial a(\theta_{K},r_{K})}{\partial \theta_{K}}\right], \end{array}$$

(49) $$\begin{array}{cc} \; & D_{r}=\left[r_{r_{1}},\ldots ,r_{r_{K}}\right]=\left[A_{2}^{*}\left(\theta ,r\right)⊙A_{2}^{\prime }\left(\theta ,r\right)+{\left(A_{2}^{\prime }\left(\theta ,r\right)\right)}^{*}⊙A_{2}\left(\theta ,r\right)\right]P, \end{array}$$

(50) $$\begin{array}{cc} \; & A_{2}^{\prime }\left(\theta ,r\right)=\left[\frac{\partial a(\theta_{1},r_{1})}{\partial r_{1}},\ldots ,\frac{\partial a(\theta_{K},r_{K})}{\partial r_{K}}\right], \end{array}$$

(51) $$\begin{array}{cc} \; & D_{\delta }=\left[r_{p_{1}^{2}},\ldots ,r_{p_{K}^{2}},r_{\sigma_{1}^{2}},\ldots ,r_{\sigma_{M}^{2}}\right]=\left[A^{*}\left(\theta ,r\right)⊙A\left(\theta ,r\right),I_{M}⊙I_{M}\right], \end{array}$$

where $I_{M}$ denotes the M×M identity matrix, and (⊙) is the Khatri–Rao product [18].

With all terms of F in Equation (36) obtained, the final CRB can now be evaluated. By applying the matrix inversion lemma [30], we arrive at the following CRBs for the location parameters θ and r, respectively:

(52) $$\begin{array}{cc} \; & {\mathrm{CRB}}_{\theta \theta }=F_{\theta \theta }^{-1}+F_{\theta \theta }^{-1}\left[F_{\theta r}\Lambda^{-1}F_{r\theta }+\Theta \Omega^{-1}\Xi \right]F_{\theta \theta }^{-1}, \end{array}$$

(53) $$\begin{array}{cc} \; & {\mathrm{CRB}}_{rr}=\Lambda^{-1}+\Lambda^{-1}\Gamma \Omega^{-1}\Upsilon \Lambda^{-1}, \end{array}$$

where

(54) $$\begin{array}{cc} \; & \Lambda =F_{rr}-F_{r\theta }F_{\theta \theta }^{-1}F_{\theta r},\;\Gamma =F_{r\delta }-F_{r\theta }F_{\theta \theta }^{-1}F_{\theta \delta }, \end{array}$$

(55) $$\begin{array}{cc} \; & \Upsilon =F_{\delta r}-F_{\delta \theta }F_{\theta \theta }^{-1}F_{\theta r},\;\Pi =F_{\delta \delta }-F_{\delta \theta }F_{\theta \theta }^{-1}F_{\theta \delta }, \end{array}$$

(56) $$\begin{array}{cc} \; & \Omega =\Pi -\Upsilon \Lambda^{-1}\Gamma ,\;\Theta =F_{\theta \delta }-F_{\theta r}\Lambda^{-1}\Gamma ,\;\Xi =F_{\delta \theta }-\Upsilon \Lambda^{-1}F_{r\theta }. \end{array}$$

## 5. Simulation Results

In this section, we conduct a series of numerical experiments with different parameter settings under an environment of nonuniform noise to evaluate the performance of the proposed algorithm. We consider a ULA structure with M=11 elements, an element spacing of d=0.4λ, and K=2 near-field targets with equal power $p^{2}$ positioned at $\left(\theta_{1},r_{1}\right)=\left(0.2^{\circ },5.1\lambda \right)$ and $\left(\theta_{2},r_{2}\right)=\left(4.2^{\circ },15.1\lambda \right)$. Except for the experiment under uniform noise in Section 5.2 and the last experiment in Section 5.5, the covariance matrix of the nonuniform noise is set to

(57) $$\begin{array}{c} Q=\mathrm{diag}\{5,3,2,1,7,4,6,8,9,0.1,0.8\}. \end{array}$$

The degree of noise imbalance is quantified by the worst noise power ratio (WNPR), defined as the ratio of the highest to the lowest noise power, with larger values indicating more severe spatial nonuniformity. We see that in (57), the WNPR is exactly 9/0.1=90. With equal signal power $p^{2}$, and the signal-to-noise ratio (SNR) is defined as [18,19]

(58) $$\mathrm{SNR}=10\log_{10}\left[\frac{p^{2}}{M}\sum_{m=1}^{M}\frac{1}{\sigma_{m}^{2}}\right].$$

We note that a ULA structure with N=11 elements and inter-element spacing d=0.4λ yields an array aperture of D=(N−1)d=4λ. The ranges of the two sources are set to r=5.1λ and r=15.1λ, both satisfying the near-field criterion

(59) $$r<\tilde{R}=\frac{2D^{2}}{\lambda }=32\lambda ,$$

where $\tilde{R}$ denotes the Rayleigh distance. The above condition r<R ensures substantial wavefront curvature across the array, leading to phase errors exceeding the π/8-radian threshold for a valid far-field approximation. Such a parameter setup guarantees significant near-field characteristics, thereby justifying the use of spherical-wave models for near-field array signal processing.

Unless otherwise specified, the above experimental conditions remain unchanged in the subsequent experiments. The results of all of the experiments are obtained by averaging 1000 independent trials, except for the simulations conducted for convergence and resolution comparison presented in Section 5.1. We adopt the root-mean-square error (RMSE) as the estimation metric. The corresponding RMSEs of the DOA estimation and the range estimation are defined as

(60) $${\mathrm{RMSE}}_{\theta }={\left[\frac{1}{1000K}\sum_{k=1}^{K}\sum_{\beta =1}^{1000}{\left({\hat{\theta }}_{k}^{\beta }-\theta_{k}\right)}^{2}\right]}^{1/2},$$

(61) $${\mathrm{RMSE}}_{r}={\left[\frac{1}{1000K}\sum_{k=1}^{K}\sum_{\beta =1}^{1000}{\left({\hat{r}}_{k}^{\beta }-r_{k}\right)}^{2}\right]}^{1/2},$$

respectively, where ${\hat{\theta }}_{k}^{\beta }$ and ${\hat{r}}_{k}^{\beta }$ represent the estimated values of $\theta_{k}$ and $r_{k}$ in the β-th trial. Besides the RMSE metric, we also use the success probability to evaluate the performance of various algorithms. The success probability for DOA estimation is defined as the proportion of cases where the absolute estimation error is less than $0.3^{\circ }$, while for range estimation, success is achieved when the estimation error is within one wavelength, i.e., λ. In addition, the search step sizes are set to $0.1^{\circ }$ for DOA and 0.1λ for range.

The proposed approach is benchmarked against the conventional MUSIC algorithm [7,31], the Capon method [27], and the reduced-dimension MUSIC (RD-MUSIC) method [17]. It is worth noting that both MUSIC and Capon require a two-dimensional search over spatial angle and range to accomplish near-field localization, which leads to relatively high computational complexity. In addition, the CRB derived in Section 4 for near-field scenarios under nonuniform noise is also included as a performance benchmark to provide a theoretical lower bound for evaluating the estimation accuracy of the proposed method.

### 5.1. Convergence and Resolution

**Figure 3:**
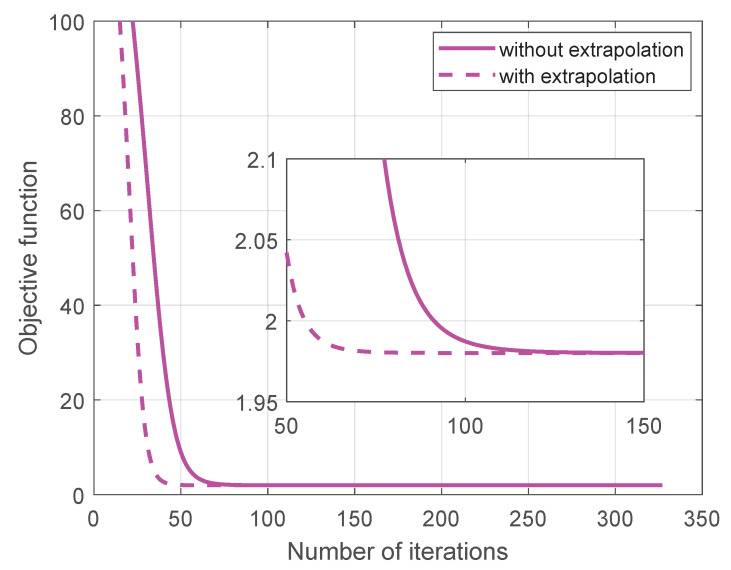
Objective function versus the number of iterations.

To examine the convergence behavior of the proposed method, we first conduct a single-run experiment involving the objective function versus the number of iterations with T=500 and SNR=10dB, as illustrated in Figure 3. It is seen that the proposed method with extrapolation for acceleration exhibits faster convergence than the one without extrapolation. In particular, the use of extrapolation enables the algorithm to reach convergence approximately 100 iterations earlier than without it, highlighting the acceleration effect of extrapolation. In the subsequent experiments, we adopt the extrapolation-enhanced version as the proposed method.

**Figure 4:**
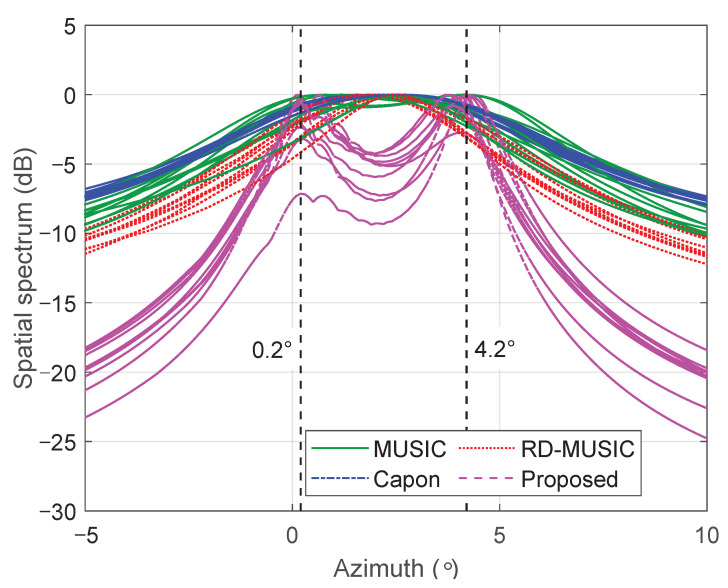
Spatial spectrum of various algorithms in 10 independent trials.

Next, we consider a spatial resolution test to evaluate the ability of different algorithms to distinguish the considered closely spaced sources at azimuth angles of $0.2^{\circ }$ and $4.2^{\circ }$, respectively. Figure 4 shows the spatial spectrum of various algorithms by averaging 10 independent trials. We see that the proposed algorithm can effectively distinguish the two signal sources, exhibiting two distinct spectral peaks at their true locations, whereas the other methods fail to do so.

### 5.2. Estimation Accuracy Versus SNR

**Figure 5:**
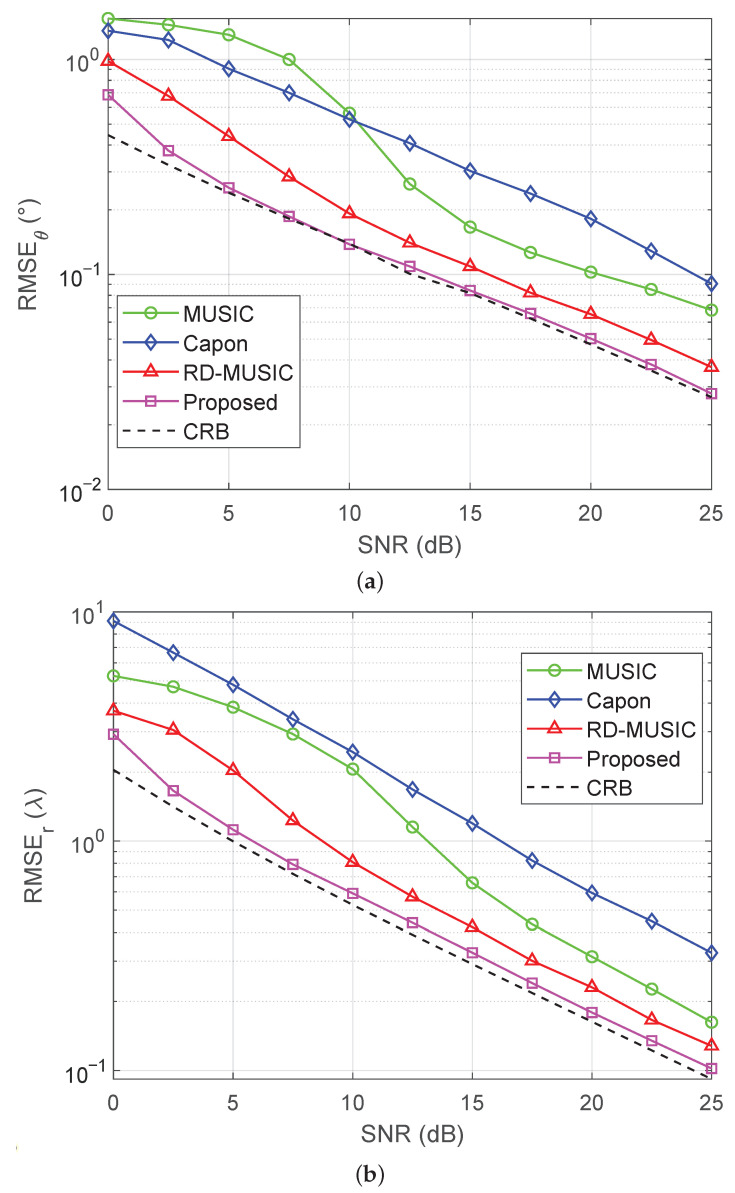
RMSEs versus SNR under nonuniform noise with M=11 and T=500: (**a**) RMSE of DOA estimation versus SNR; (**b**) RMSE of range estimation versus SNR.

**Figure 6:**
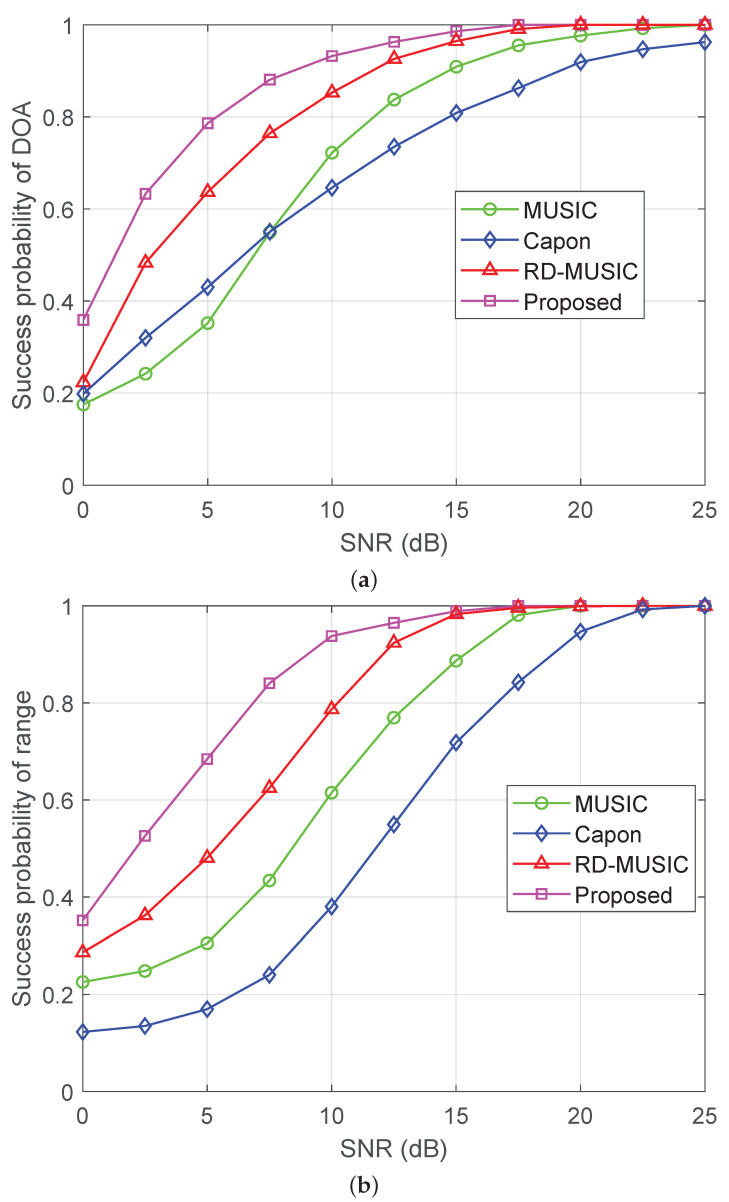
Success probabilities versus SNR under nonuniform noise with M=11 and T=500: (**a**) success probability of DOA estimation versus SNR; (**b**) success probability of range estimation versus SNR.

Figure 5a,b show the RMSEs of DOA estimation and range estimation versus SNR with M=11 sensors and T=500. We see that the proposed method consistently attains the lowest RMSE across all algorithms and closely approaches the CRB at a relatively high SNR, which indicates its consistent effectiveness. However, the Capon method performs poorly, primarily due to its incompatibility in environments of nonuniform noise. The performance loss of MUSIC and RD-MUSIC arises from subspace estimation errors introduced by eigendecomposition of the sample covariance matrix in the presence of nonuniform noise. In addition, the RD-MUSIC algorithm achieves separation of angle and distance parameters through dimensionality reduction. As such, it mitigates the error interference caused by the coupling of the two parameters in the near-field two-dimensional search, and therefore performs slightly better than MUSIC. Figure 6a,b depict the success probabilities of various algorithms versus the SNR. A similar performance trend is observed as in the RMSE versus SNR results shown in Figure 5. It is clear that the proposed algorithm maintains the highest success probability throughout the entire SNR range, approaching unity when the SNR exceeds 15 dB.

**Figure 7:**
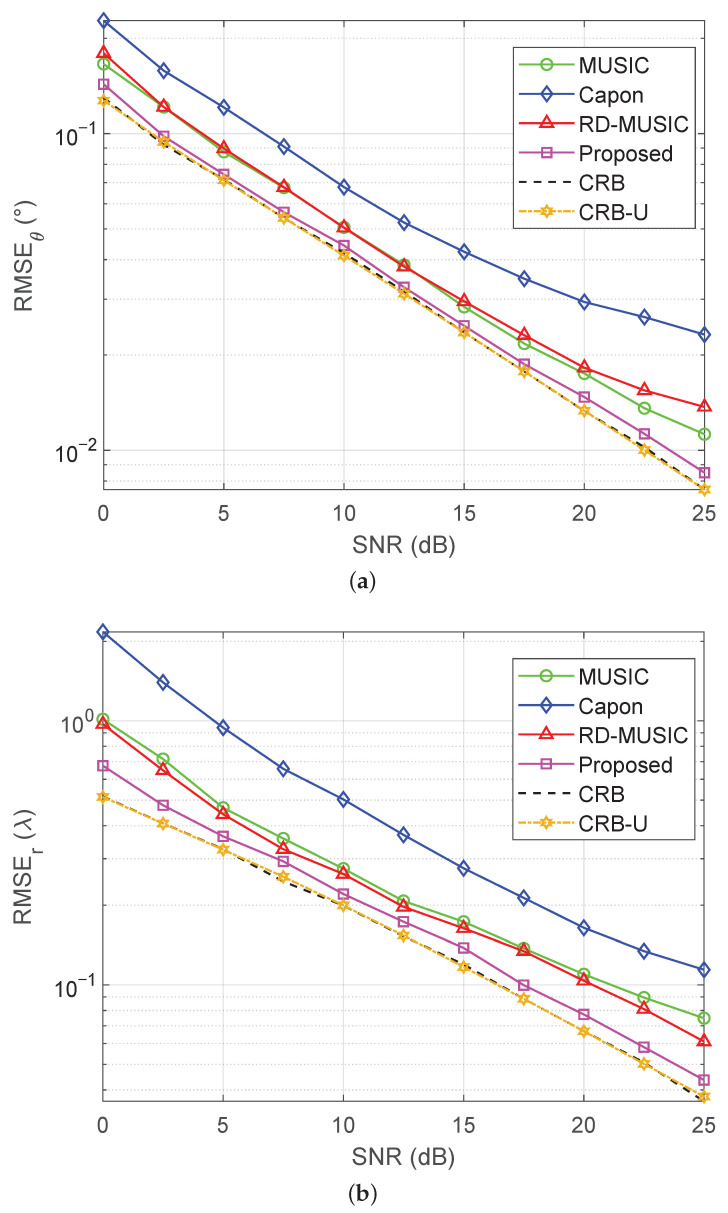
RMSEs versus SNR under uniform noise with M=11 and T=500: (**a**) RMSE of DOA estimation versus SNR; (**b**) RMSE of range estimation versus SNR.

We now consider the uniform-noise case, where all diagonal elements of Q are set to one, in order to test the adaptability of the proposed algorithm under uniform-noise conditions. The estimation accuracy of the DOA and the range versus the SNR are depicted in Figure 7. It is observed that the CRB derived in this work is consistent with the CRB-U reported in [32] under near-field uniform-noise conditions. At the same time, the proposed algorithm achieves the best performance among all baseline localization methods and closely approaches both CRBs.

### 5.3. Estimation Accuracy Versus the Number of Snapshots

**Figure 8:**
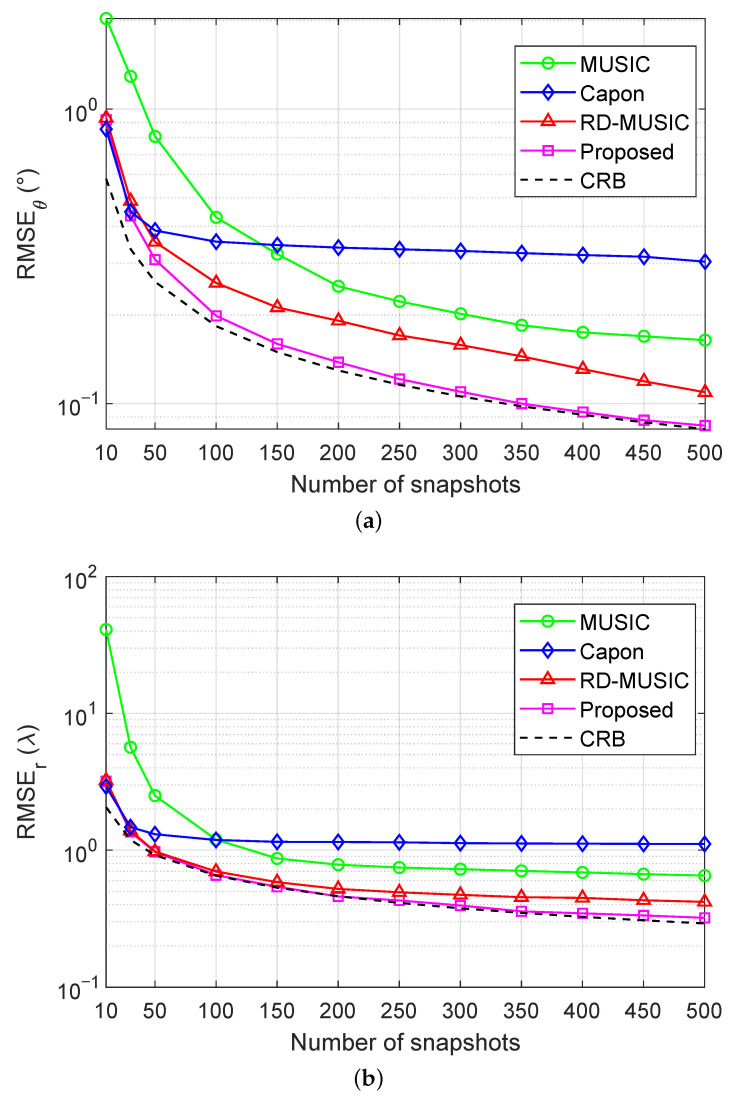
RMSEs versus snapshots under nonuniform noise with M=11 and SNR = 15 dB: (**a**) RMSE of DOA estimation versus the number of snapshots; (**b**) RMSE of range estimation versus the number of snapshots.

We now consider the test of estimation accuracy versus the number of snapshots. Figure 8a,b depict RMSEs against the number of snapshots with SNR=15 dB. It is seen that the proposed method generally performs better in terms the RMSEs of DOA estimation and range estimation as the number of snapshots increases. Furthermore, throughout the entire evaluation range of the number of snapshots, the RMSEs of the proposed method remain close to the CRB. Particularly, the RMSEs of both the DOA and range of the proposed method begin to approximate the CRB when the number of snapshots exceeds 100. In contrast, when the number of snapshots is small, MUSIC exhibits particularly notable errors, with RMSEs far from those of the CRB. These results indicate that the proposed algorithm exhibits excellent estimation performance, with asymptotic efficiency approaching the CRB for large sample sizes.

**Figure 9:**
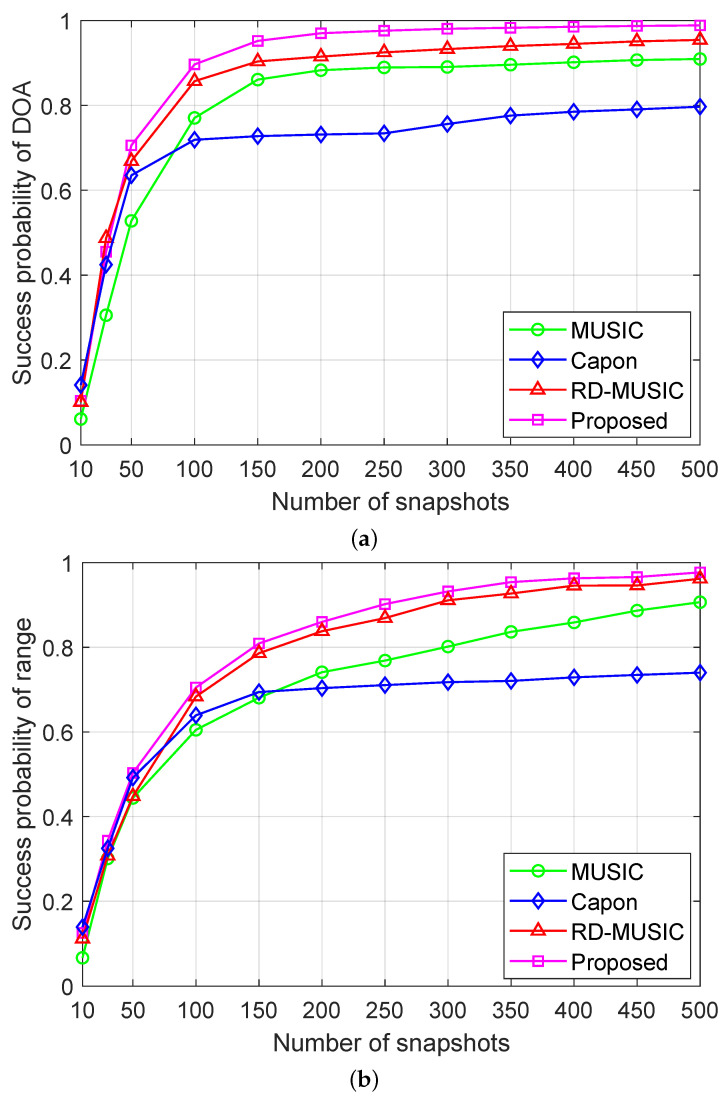
Success probabilities versus the number of snapshots under nonuniform noise with SNR = 15 dB and M=11: (**a**) success probability of DOA estimation versus the number of snapshots; (**b**) success probability of range estimation versus the number of snapshots.

Figure 9a,b show the success probabilities of the respective algorithms versus the number of snapshots. We see that the success probability of all algorithms increases monotonically with an increasing number of snapshots, and the proposed method exhibits the best performance. In particular, when the number of snapshots is 100, the proposed algorithm achieves a success probability exceeding 0.9, while the other compared methods require more snapshots or cannot reach this level. These results validate the significant performance advantages of the proposed algorithm in the near-field nonuniform-noise environment.

### 5.4. Estimation Accuracy Versus the Number of Sensors

**Figure 10:**
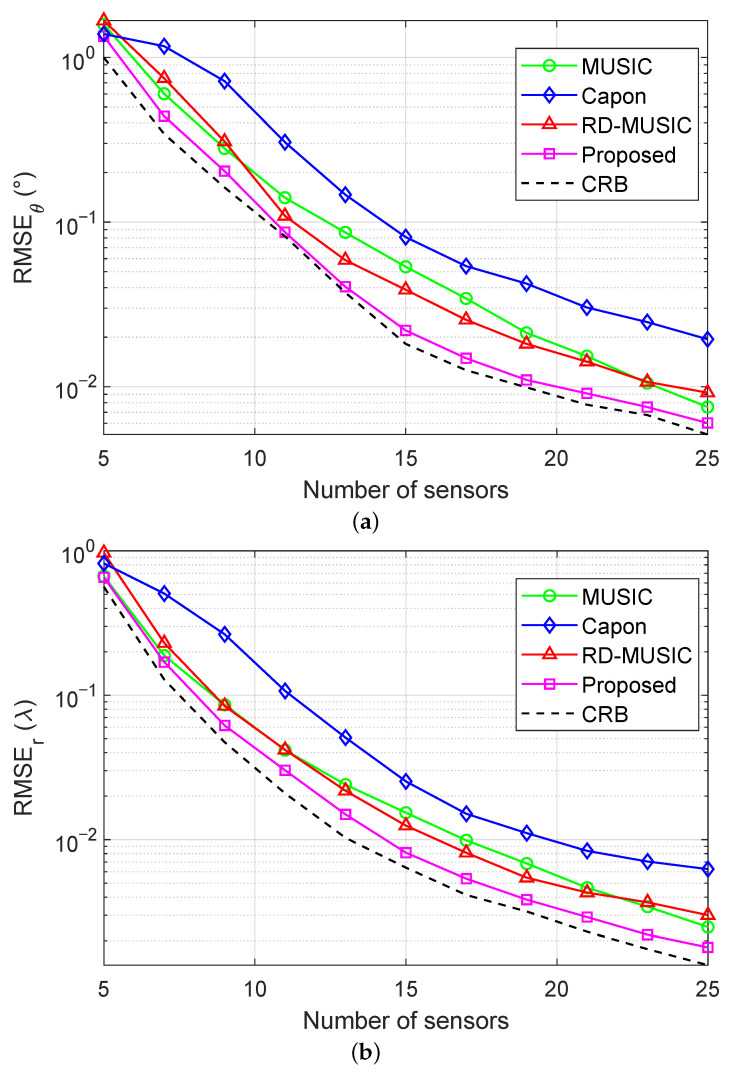
RMSEs versus the number of sensors under nonuniform noise with SNR = 15 dB and T=500: (**a**) RMSE of DOA estimation versus the number of sensors; (**b**) RMSE of range estimation versus the number of sensors.

We depict the RMSEs of DOA estimation and range estimation versus the number of sensors, varied from 5 to 25, in Figure 10a,b, respectively, under the conditions of SNR=15 dB and T=500. We see that as the number of sensors increases, the estimation performance of all algorithms improves considerably. This improvement is attributed to the extended array aperture provided by additional sensors. Moreover, the RMSEs of the proposed method closely match those of the theoretical CRB, indicating its near-optimal estimation accuracy. In contrast, the traditional MUSIC and Capon estimators yield significantly larger errors, while RD-MUSIC, although improved, still falls notably short of the proposed method.

**Figure 11:**
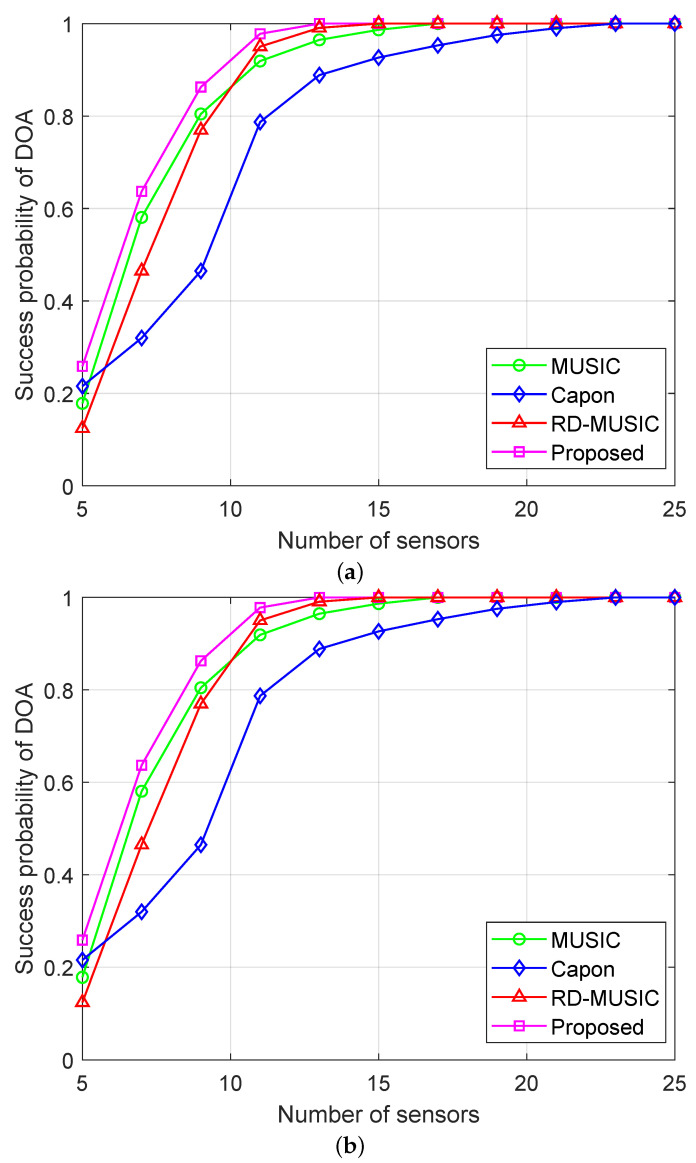
Success probabilities versus the number of sensors under nonuniform noise with SNR = 15 dB and T=500: (**a**) success probability of DOA estimation versus the number of sensors; (**b**) success probability of range estimation versus the number of sensors.

Figure 11a,b illustrate the success probabilities of DOA estimation and range estimation as a function of the number of sensors, respectively. We observe that although the performance of all algorithms improves with an increase in the number of sensors, the proposed algorithm consistently outperforms the other benchmark methods. Notably, to achieve a probability of approximately 90% in DOA estimation, the proposed method requires only 9 sensors, whereas the next-best RD-MUSIC achieves only a probability of about 75% with the same conditions. This advantage is also evident in range estimation. In addition, the proposed algorithm achieves a probability of 100% with approximately 13 sensors, whereas the same probability of success can only be achieved by the other methods with 15 or more sensors.

### 5.5. Estimation Accuracy Versus WNPR

**Figure 12:**
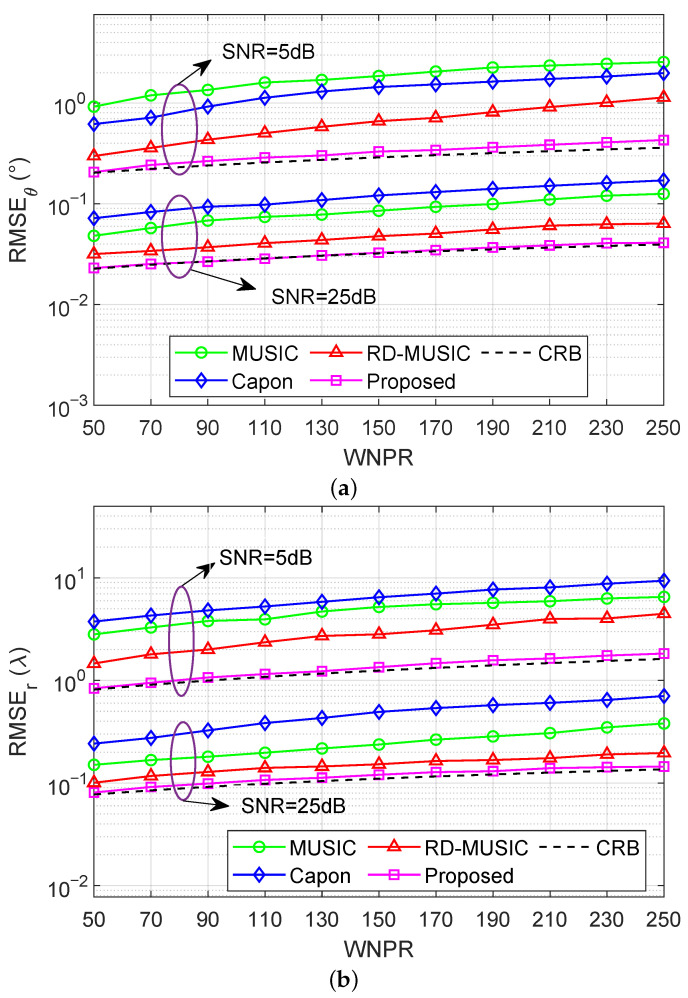
RMSEs versus WNPR under nonuniform noise with M=11, K=2, T=500, and SNR∈{5dB,25dB}: (**a**) RMSE of DOA estimation versus WNPR; (**b**) RMSE of range estimation versus WNPR.

To further evaluate the robustness of the proposed approach against nonuniform noise, we plot the RMSEs of various algorithms against the WNPR, with T=500, in Figure 12. In this case, the WNPR is varied from 50 to 250 in steps of 50. Meanwhile, we consider two cases of SNRs with SNR=5 dB and SNR=25 dB. It is seen that, for both cases with DOA estimation in Figure 12a and range estimation in Figure 12b, all methods exhibit monotonic RMSE growth with increasing WNPR, consistent with our expectation that degradation occurs under stronger nonuniform noise. Moreover, the RMSE of the proposed algorithm closely follows that of the CRB throughout the entire WNPR range, demonstrating clear superiority over the other compared methods, which exhibit a certain sensitivity to noise imbalance. These results demonstrate that the proposed algorithm achieves near-optimal performance under a favorable SNR while also maintaining robustness and accuracy in the presence of severe nonuniform noise.

### 5.6. Computation Time Comparison

**Figure 13:**
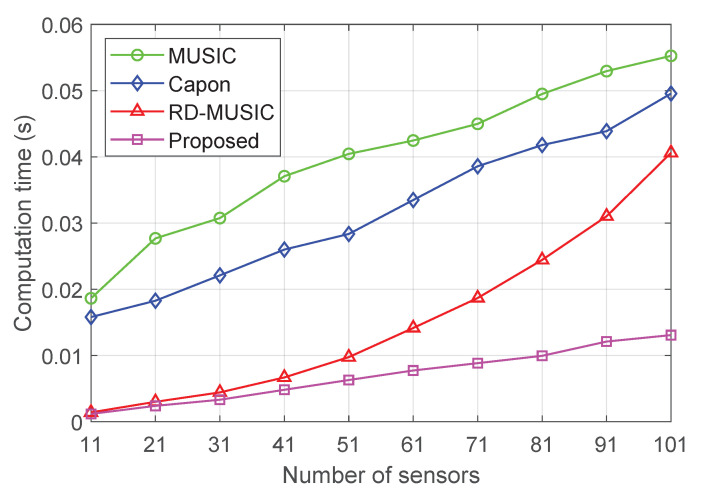
Computation time of various algorithms versus the number of sensors.

Finally, we compare the running time of various algorithms to evaluate the computational efficiency of the proposed method. Figure 13 shows the computation time of various algorithms versus the number of sensors, ranging from 11 to 101 with a step of 10, with the parameters T=500 and SNR=15 dB. We see that the proposed algorithm demonstrates significantly superior computational performance the over other compared methods and keeps the running time below 0.02 s across all the considered number of sensors. Particularly, the computation time of the proposed method increases approximately linearly with the number of sensors, indicating good scalability in terms of computational cost. In contrast, the computation times of MUSIC, Capon, and RD-MUSIC grow at a much faster rate, especially for large-scale arrays.

## 6. Conclusions

In this paper, we investigated the problem of near-field source localization under a nonuniform-noise environment. Specifically, we proposed a symmetric matrix factorization framework with unknown noise powers to be estimated for fitting a sample covariance matrix. Meanwhile, we developed a computationally efficient algorithm to jointly estimate the symmetric matrix factor and the noise covariance matrix by using the block majorization–minimization principle. Each iteration of the proposed algorithm yielded a closed-form update, enabling accurate recovery of the signal subspace. In addition, the stochastic CRB for near-field localization under nonuniform noise was also derived. Our extensive numerical experiments confirm that the proposed method delivers high estimation accuracy and computational efficiency, with its performance closely approaching that of the CRB.

## Figures and Tables

**Table 1:** Computational complexity comparison.

Algorithm	Complexity
Proposed	$O\left(n_{g}\left(M+1\right)\left[\frac{\left(M-K)(3M+1\right)}{4}+\frac{{\left(M+1\right)}^{2}}{8}\right]+W_{\max}\left[MK^{3}+M^{2}\left(K^{2}+T\right)+M\right]\right)$
RD-MUSIC	$n_{g}\left(M+1\right)\left[\left(M-K\right)\left(3M+1\right)/4+{\left(M+1\right)}^{2}/8\right]+M^{3}+M^{2}T$
Capon	$TM^{2}+\frac{2}{3}M^{3}+N_{s}\left(M^{2}+2M\right)$
MUSIC	$\left(n_{g_{\theta }}n_{g_{r}}\right)\left(M-K\right)\left(M+1\right)\left(3M+1\right)/4+M^{3}+M^{2}T$

## Data Availability

No new data were created or analyzed in this study. Data sharing is not applicable to this article.
